# Effects of Fish Oil Supplementation on Pregnancy Outcomes in Pregnant Women Referred to Kosar Hospital

**DOI:** 10.22037/ijpr.2019.13976.12041

**Published:** 2020

**Authors:** Nazanin Gholami, Shokoh Abotorabi, Fatemeh Lalooha, Sonia Oveisi

**Affiliations:** a *Department of Obstetrics and Gynecology, Qazvin University of Medical Sciences, Qazvin, Iran. *; b *Department of Obstetrics and Gynecology, Clinical Research Development Unit, Kosar Hospital, Qazvin University of Medical Sciences, Qazvin, Iran. *; c *Department of Maternity and Child Health, Metabolic Disease Research Center, Qazvin University of Medical Sciences. Qazvin, Iran.*

**Keywords:** Fish Oils, Fatty Acids, Omega-3, Pregnancy Outcome, Preterm birth, Infant

## Abstract

The hypothesis of a protective effect of fish oil supplementation in preventing some consequences of pregnancy such as gestational hypertension is put forward which has attracted increasing attention. The aim of the present study was to evaluate the effect of fish oil supplementation on outcomes of pregnancy. This study was a clinical trial performed on 339 women with singleton pregnancy aged 18-35 and gestational age of 20 weeks who visited prenatal clinic at Kosar Hospital in Qazvin during 2015-2016. Patients were randomly divided into two groups marked as intervention group which received soft gelatin capsules (each containing 1000 mg fish oil including 120 mg DHA and 180 mg EPA) on a daily basis from the 20^th^ week to the end of pregnancy, and the women in the control group with no fish oil intake. The outcomes of pregnancy including preeclampsia, eclampsia, preterm labor, gestational diabetes, weight, height, head circumference at birth and the gestational age at delivery were evaluated in both groups. Data were analyzed using statistical tests including Mann-Whitney U test and *t*-test. There was significant difference in gestational age between the two study groups (*P* < 0.05). There was no significant difference in the percentage of preterm birth, preeclampsia, eclampsia, IUGR, and GDM between the two groups (*P *> 0.05). The results of this study showed that consumption of fish oil supplements from 20^th^ week of gestation by 18-35 year-old pregnant women increased pregnancy age but failed to decrease the percentage of preterm birth, preeclampsia, eclampsia, IUGR, and GDM.

## Introduction

The nutritional status of mother before and during pregnancy is a determination factor associated with morbidity before and during childbirth, weight at birth, and other parameters related to appropriate feeding (1)**.** Nutritional recommendations for mothers emphasize over the consumption of essential proteins, vitamins, and minerals nevertheless, in recent years the inclusion of long-chain unsaturated omega-3 fatty acids in the diet are also strongly suggested (2, 3). These essential fatty acids should be received through foods or food supplements as the human body can not synthesize such nutrients (4). Biologically, docosahexaenoic acid (DHA) and eicosapentaenoic acid (EPA) are the most active long-chain unsaturated omega-3 fatty acids abundantly found in fish oil (5-7). Consumption of DHA and its concentration in mother’s blood flow is an important factor affecting the DHA level in fetus blood circulation since during pregnancy DHA is transported to fetus through placenta and the speed of transport is higher during the last trimester of pregnancy due to faster growth rate of fetus (6, 8-11).

Evaluating the effect of long-chain unsaturated fatty acids found in fish oil has been the focus of many recent studies due to the availability of some evidence on improved pregnancy outcomes including gestational diabetes, preeclampsia, fetus growth, prolonged gestational age, weigh, height, and head circumference of infant (2, 12, 13). 

In some studies, it is reported that the consumption of fish oil as antioxidant has no effect on reducing the incidence of preeclampsia whereas in a numer of other studies the results mentioned are quite the opposite (14-18). Also, another hypothesis suggests that increased consumption of fish oil rich in long-chain unsaturated fatty acids could delay the time for spontaneous labor which is probably due to the effect of these fatty acids on the prostaglandins involved in the initiation of parturition and its further action on myometrial activity (19). When the findings of several studies revealed increase fetus growth and prolonged gestational age following consumption of fish oil, many researchers started to investigate the role of omega-3 fatty acids in particular EPA and DHA in pregnancy although the convincing evidence regarding the randomized clinical trials were both limited and with conflicting results (20-24).

The known effects of fish oil are attributed to DHA and EPA. The aim of fish oil consumption is to receive DHA and EPA which are both available in fish oil-derived products. These two omega-3 fatty acids are of prime importance for the human body as DHA is the most common fatty acid of the human brain and responsible for final development of nervous system (25, 26).

Preeclampsia is one of the most common complications observed in the course of pregnancy and occurs in 5-10% of all pregnancies and 20% of the first pregnancies and is the cause of more than 40% of early labor following treatment. Omega-3 fatty acids cause an increase in vascular permeability and vasodilation leading to reduced blood pressure (27-30). 

Considering the controversy over the role of fatty acids in the form of fish oil and its consumption during pregnancy and as the public health programs and policies at national and regional levels in developing countries should be designed in a way to boost and improve the quality of care in pregnancy, the present study was performed to investigate the effect of fish oil supplementation on the outcomes of pregnancy including the incidence of preeclampsia, eclampsia, preterm labor, gestational age at delivery, birth weight and height, and the occurrence of gestational diabetes in two case and control groups. 

## Experimental

This was a clinical trial (IRCT2015072123269N1) approved by the Ethics Committee of Qazvin University of Medical Sciences (ID number: IR.QUMS.REC.1394.234). The objective of the study was clearly explained for all pregnant women aged 18-35 who visited the prenatal clinics of Kosar training hospital from November 2015 to April 2016 in Qazvin and were fit to enter the study. 

*Inclusion criteria*

Inclusion criteria include pregnant women, age 18-35, gestational age of 20 weeks, fifth pregnancy or lower, and a singleton pregnancy.

*Exclusion criteria*

 Exclusion criteria were smoking, drug addiction, eating fish more than twice a week, hemorrhagic diseases, anti-coagulation therapy, use of anti-hypertensive drugs, BMI>30, history of allergy to fish products, presence of chronic hypertension, preeclampsia, eclampsia, history of early labor, known underlying disease such as cardiac, renal, gastrointestinal, pulmonary, thyroid or autoimmune diseases, diabetes, impaired glucose tolerance test at the beginning of current pregnancy, epilepsy, and secondary high blood pressure.

All participants submitted written consent form and then a simple random sampling was used and the subject were divided into 2 groups of case (fish oil) and control no drug. A total of 180 pregnant women were examined in each group and the pregnancy care delivered to both groups was similar to the hospital routine care offered to all pregnant women and included regular examinations by a gynecology resident every 4 weeks until 28 week, every 2 week between 28-36 weeks, and then every week on wards to the end of pregnancy.

The women in the case group received soft gelatin capsules each containing 1000 mg fish oil (made by Zahravi company) including 120 mg DHA and 180 mg EPA on a daily basis starting from the week 20 to the end of pregnancy, and those in the control group with no fish oil intake. The outcomes of pregnancy including the occurrence of preeclampsia, eclampsia, early delivery, gestational diabetes in mothers and weight, height, and head circumference of babies at birth as well as the gestational age at labor were evaluated. The total number of fish oil capsules required for each course was delivered to all members of the case group after each visit session. All participants were followed up until delivery to determine the gestational age, weight, and height at birth and the incidence of preeclampsia, eclampsia, preterm labor, and gestational diabetes. This research was performed according to the Declaration of Helisinki i.e. ethical principles for medical research, following the approval of the project by the Ethic Committee of Qazvin University of Medical Sciences. Patients were fully informed of the details of the research through delivery of adequate explanation over the method and the objective of the study. All patients provided written consent form before entry to the study. The personal information of all participants was kept private and only the results of the survey were published. 

*Statistical Analysis*

Demographic information of patients including age, gravidity, education level, income, and other variables under the study were collected in a checklist, specifically designed for this study, and the data were further analyzed by computer statistical tool (SPSS version21) using Mann-Whitney U-test and *t*-test. A *P*-value less than 0.05 was considered as significant, statistically. 

*Limitations*

As the pregnant women had to visit the prenatal clinic at Kosar training hospital to get pregnancy care, some parameters such as migration of patient, physician change, and so on could be regarded as the limitations of the study.

## Results

In the present study, the number of participants who continued to be part of the survey until delivery was 179 and 160 pregnant women in the case and control groups, respectively. Parity between 2 groups (nullipar and multipar) had significant difference (Table 1).

In the present survey, the mean gestational age at labor was 38.4 ± 1.8 weeks and 2.6 ± 2.3 days in the control group and 38.9 ± 1.7 weeks and 3.3 ± 2.2 days in the case group and the difference was significant, statistically (*p *< 0.05). The values obtained for the gestational age at delivery (week) in two study groups revealed significant difference, statistically (*p *< 0.05). The gestational age in the case group was higher than that found for the control group. Finally, no significant difference between the two study groups regarding the weight, height, and head circumference of babies at birth was established (*p *> 0.05) (Table 2). Demographic data includes the mean weight, height and circumference of babies at birth (Table 3). 

## Discussion

The final objective of the current study was to investigate the impact of fish oil supplementation on pregnancy outcomes in pregnant women who visited the prenatal clinics of Kosar training hospital in Qazvin. Since the majority of differences associated with the interfering variables (covariates) such as age of mothers, BMI, and number of previous delivery and abortion were not found to be significant between the two case and control groups (*p *> 0.05), therefore the results obtained in this study could be, to some extent, reliable.

In the present survey, the mean gestational age at labor was 38.4 ± 1.8 weeks and 2.6 ± 2.3 days in the control group and 38.9 ± 1.7 weeks and 3.3 ± 2.2 days in the case group and the difference was significant, statistically (*p *< 0.05). The gestational age at delivery in the case group was 4 days longer than that observed in the control group. This finding is in agreement with the results reported by Smuts *et al.* .who showed that the gestational age at delivery increased by 6 ± 2.3 days in the case group, compared to the control group, after inclusion of 133 mg fish oil to their regular daily diet starting from 24-28 weeks until childbirth (31). 

There was no significant difference between the two groups associated with the percentage of preterm labor, preeclampsia, eclampsia, IUGR, and GDM (*p *> 0.05) however, the percentage values found for these items in the case group were lower compared to the control group. Olsen *et al* in their study on the impact of fish oil supplementation in a high-risk pregnancy showed the consumption of fish oil supplements had no effect on IUGR and pregnancy-associated HTN either in singleton or twin pregnancy (16). Likewise, Onwude *et al*. examined the effect of fish oil in high-risk pregnancy including cases of proteinuria, pregnancy-associated HTN, and IUGR, and found no significant difference in the level of blood pressure in pregnant women with proteinuria-induced HTN and those with non-proteinuric pregnancy-induced HTN (17). In another study by Salvig *et al*., investigating the effect of fish oil supplement on blood pressure in pregnancy, the authors mentioned there was no statistically significant difference in systolic or diastolic pressure in the group with fish oil supplement intake and the two other groups used in their study (18). Also, Krauss-Etschmann *et al*. evaluated the effect of fish oil supplements and folate on the maternal and fetal plasma concentration of eicosapentaenoic acid (EPA) and docosahexaenoic acid (DHA) and concluded that there was no significant difference in pregnancy outcomes and fetus growth between their study groups (23).

The administration of supplements containing antioxidants and essential fatty acids (DHA and EPA) during pregnancy may be considered as an approach to fighting oxidative stress and eventually preventing or delaying the initiation of preeclampsia, leading to improvement in the health status of both mother and infant (31). 

It is believed that omega-3 polyunsaturated fatty acids (n-3 PUFAs) increase the ratio of prostacyclins to thromboxanes, dilute the blood, and increase the flow of fetal blood perfusion. That’s the issue that could facilitate and improve the growth of the fetus and also reduced the production of the pro-inflammatory n-6 AA derived prostaglandins and increased the length of pregnancy. (32)

As described above, the results of some studies are compatible with those found in the current study and some in disagreement with the findings obtained in the present study. There are various explanations for this discrepancy. The most important reason for this inconsistency between this study and those of others could be due to the type of fish oil and also the way it was consumed. In some studies, fish oil was used as a liquid oil, fish oil capsules, taken directly from fish, or omega-3 fatty acids.

The second important and affecting factor that could interfere with the findings in various studies is due to the dose or quantity of fish oil supplement used in different experiment. The third influencing parameter was the timing of fish oil supplement consumption by pregnant women which was different in various studies as the consumption of fish oil started from the beginning of pregnancy in some studies whereas others began to use fish oil at mid-pregnancy i.e. From the 22^nd^ week of gestation onward to the end of gestation, which certainly affect the findings of the study. Other factors such as mother’s age, type of daily activities, occupation, ecological condition of location for clinical examinations, type of human race, and the type of study all influence the results of studies. Finally, the method used for statistical analysis is also among the affecting factors that may change the findings obtained in different studies. In some studies *t*-test was used to compare the means obtained for both case and control groups while in others attention was only paid to determine a correlation between the variables and this, despite the presence of significant difference between the dependent and independent variables, will not lead to significant difference between the case and control groups. 

In conclusion, the results of the present study indicated that consumption of fish oil supplement by pregnant women aged 18-38 and at gestational age of 20 weeks led to prolonged gestational age at labor but with no effect on percentage of preterm labor, preeclampsia, eclampsia, IUGR, and GDM. Also, fish oil supplementation during pregnancy produced no significant effect on infant’s parameters such as weight, height, and head circumference at birth. There are some suggestions that could be followed by other researchers for future studies. These recommendations are investigating the effect of different methods for consuming fish oil including fish oil supplement as capsules, natural fish oil, and fish dishes on pregnancy outcomes and further comparison of results; examining the effect of fish oil supplementation on different stages of pregnancy; comparing different time intervals for fish oil supplementation during pregnancy; and eventually evaluating the anti-oxidant effect of fish oil supplementation on maternal and fetal indices. 

**Table 1:** Demographic characteristics of the 2 groups

		**Case group**	**Control group**	***P*** **-value**
		**N = 160**	**N = 179**	
Parity^*^	nullipar	99(61.9)	79(44.9)	0.002
	multipar	61(38.1)	97(55.1)	
Age^**^		26.5 ± 4.6	26.8 ± 5.1	0.547
Weight^**^		64.8 ± 8.5	66.5 ± 8.2	0.063
Height^**^		161.6 ± 5.3	161.3 ± 5.6	0.609
BMI^**^		24.8 ± 3.2	25.5 ± 2.8	0.030

^*^Data are present as N (%).

^**^Data are present as mean ± SD.

**Table 2:** Comparison of the variables associated with labor in two case and control groups

	**Case group**	**Control group**	***P*** **-value**
	**N = 160**	**N = 179**	
Gestational Age at labor (week)^*^	38.9 ± 1.5	38.4 ± 1.8	0.019
Preterm Labor^**^	7 (4.4)	17 (9.5)	0.089
Preeclampsia^**^	4 (2.5)	9 (5.0)	0.268
Eclampsia^**^	0	0	-
IUGR^**^	0	3 (1.7)	0.250
GDM^**^	9 (5.6)	14 (7.8)	0.518

^ *^Data are present as mean ± SD.

^ **^Data are present as N (%).

**Table 3:** The variables associated with the infants at birth in two case and control groups

	**Case group** **N = 160** **Mean ± SD**	**Control group** **N = 179** **Mean ± SD**	***P*** **-value**
Weight Baby	3184.1 ± 332.4	3138.5 ± 471.7	0.302
Height Baby	48.0 ± 2.7	48.0 ± 2.4	0.976
Head Circumference	36.7 ± 2.3	36.7 ± 1.9	0.883
